# SIRT3/6/7: promising therapeutic targets for pulmonary fibrosis

**DOI:** 10.3389/fcell.2025.1557384

**Published:** 2025-04-02

**Authors:** Pingping Huang, Dan Qin, Yanling Qin, Sha Tao, Guangnan Liu

**Affiliations:** ^1^ Department of Respiratory and Critical Care Medicine, The Second Affiliated Hospital of Guangxi Medical University, Nanning, China; ^2^ Department of Endocrinology, The Second Affiliated Hospital of Guangxi Medical University, Nanning, China

**Keywords:** sirtuins, pulmonary fibrosis, mechanism, inflammation, signaling pathways, EMT

## Abstract

Pulmonary fibrosis is a chronic progressive fibrosing interstitial lung disease of unknown cause, characterized by excessive deposition of extracellular matrix, leading to irreversible decline in lung function and ultimately death due to respiratory failure and multiple complications. The Sirtuin family is a group of nicotinamide adenine dinucleotide (NAD+) -dependent histone deacetylases, including SIRT1 to SIRT7. They are involved in various biological processes such as protein synthesis, metabolism, cell stress, inflammation, aging and fibrosis through deacetylation. This article reviews the complex molecular mechanisms of the poorly studied SIRT3, SIRT6, and SIRT7 subtypes in lung fibrosis and the latest research progress in targeting them to treat lung fibrosis.

## 1 Pulmonary fibrosis

Among the numerous causes of pulmonary fibrosis include autoimmune disorders, industrial or environmental exposures, adverse drug reactions, and numerous other conditions. An underlying cause is unknown for idiopathic pulmonary fibrosis (IPF), a chronic progressive fibrosing interstitial lung disease. It is considered to be the fastest progressing and most lethal of all fibrotic diseases (Lederer and Martinez, 2018; Mei et al., 2021; Qian et al., 2021). Its main feature is the excessive deposition of extracellular matrix (ECM), which leads to irreversible loss of lung function and collapse of lung tissue structure, accompanied by progressive scarring, and ultimately death due to respiratory failure and various complications. IPF mainly affects middle-aged and elderly people. According to statistics, the average survival time of untreated IPF patients after diagnosis is only 3–5 years (Raghu et al., 2018). Globally, the number of people with IPF is estimated to exceed 3 million. According to epidemiological research, the incidence and prevalence of IPF range from 0.09 to 1.30 per 10,000 individuals and are rising annually. The incidence of IPF is comparatively high in the US, South Korea, and Canada as compared to other nations (Schäfer et al., 2020; He et al., 2021; Maher et al., 2021). The pathological mechanism of IPF includes an initial diffuse inflammatory response, proliferation of mesenchymal cells, excessive deposition of ECM and formation of fibrosis. Persistent inflammation and abnormal repair mechanisms are key factors driving the progression of IPF (Quan et al., 2020). Currently, there is still no effective treatment for IPF. While pirfenidone and nintedanib, two medications licensed by the FDA for the treatment of IPF (Shenderov et al., 2021), they can only slow the progression of the disease and the deterioration of lung function, and cannot reverse the lung damage that has already occurred.

Lung transplantation is currently the only clinically proven effective treatment, but due to its high cost, the scarcity of donors, and the risk of rejection after surgery, the clinical application of lung transplantation is very limited (Han et al., 2022). Therefore, the management goals of IPF mainly focus on symptomatic treatment, improving patients’ quality of life, maintaining lung function, and prolonging patients’ survival as much as possible (Olson et al., 2018).

## 2 Sirtuins family

The sirtuin family is a subclass of class III histone deacetylases (HDACs), which are dependent on nicotinamide adenine dinucleotide (NAD+). This family includes seven members, SIRT1 to SIRT7, which are widely involved in various biological activities in the body by deacetylation of multiple substrates (Bordone and Guarente, 2005; Michan and Sinclair, 2007). The deacetylating effect of sirtuins regulates the conformation of proteins and their interactions with other molecules by removing the acetyl group from proteins and changing the surface charge distribution, thereby regulating the conformation of proteins and their interactions with other molecules, and participating in the regulation of multiple cellular processes, including transcriptional regulation, cell proliferation and division, DNA damage repair, metabolic regulation, inflammatory response, apoptosis and aging, oxidative stress, and mitochondrial biogenesis (Chen et al., 2015a; Yan et al., 2017; Chu et al., 2018; Alves-Fernandes and Jasiulionis, 2019; de Gregorio et al., 2020; Prabhakar et al., 2020). The sirtuins family is an important epigenetic regulatory factor. The deacetylation mediated by them is one of the most prominent epigenetic mechanisms. That is, sirtuins reverse the action of acetyltransferases by removing the acetyl group from lysine residues (Imai et al., 2000). Studies have shown that SIRT3 can inhibit FOS transcription by deacetylating histone 3 lysine 27 (H3K27), reducing inflammation and fibrosis (Palomer et al., 2020). SIRT6 can promote the destabilization of the chromatin of the target gene RELA and the termination of NF-κB signaling by deacetylating histone H3 lysine 9 (H3K9), affecting inflammatory and metabolic processes (Kawahara et al., 2009). In addition, SIRT7 regulates the histone H3 lysine 18 site (H3K18) deacetylation of Dicer in the DNA damage response, revealing the role of SIRT7 in DNA damage repair (Zhang et al., 2016). The role of the sirtuin family in fibrotic diseases has attracted much attention. Studies have shown that sirtuins play a role in the onset of fibrosis in many types and organs, and are considered to be endogenous regulators of fibrogenesis during the development of lung fibrosis. It has been confirmed that SIRT1, SIRT2, SIRT3, SIRT6 and SIRT7 significantly inhibit the development of lung fibrosis (Bindu et al., 2017; Wyman et al., 2017; Chu et al., 2018; Zhang et al., 2019b; Gong et al., 2021). Furthermore, numerous carefully planned investigations have demonstrated the significant protective effect sirtuins play in anti-cardiovascular and liver fibrosis. However, compared with cardiovascular and liver fibrosis, studies on Sirtuins in pulmonary fibrosis are still relatively rare, especially those on SIRT3, SIRT6 and SIRT7. This provides a broad research space to explore their functions and mechanisms in pulmonary fibrosis.

This review focuses on the role of SIRT3, SIRT6, and SIRT7 in fibrotic diseases, analyzes their molecular regulatory mechanisms in pulmonary fibrosis, and summarizes the latest research progress for the therapy of IPF using SIRT3, SIRT6, and SIRT7 as targets.

## 3 Molecular mechanism of sirtuins 3/6/7 regulating pulmonary fibrosis

ECM protein deposition and lung fibroblast and myofibroblast proliferation are hallmarks of lung fibrosis (Mayr et al., 2024). Damaged alveolar epithelial cells (AECs) can induce the secretion of inflammatory factors by related inflammatory cells, which further stimulate the transformation of AECs into lung fibroblasts, accelerate the process of epithelial-mesenchymal transition (EMT), and promote fibroblast-to-myofibroblast transition (FMT). Myofibroblasts secrete large amounts of collagen, which promotes the progression of pulmonary fibrosis. Increased cross-linking of the extracellular matrix (Burgess et al., 2024), dysregulation of matrix metalloproteinases and resistance of fibroblasts to apoptosis may lead to increased alveolar septum thickness, fibrotic destruction of normal lung structure, loss of gas exchange, and ultimately severe respiratory failure and death (Islam et al., 2023; Chen et al., 2024c).

SIRT7 is one of the least studied members of the Sirtuins family. SIRT7 is located in the nucleolus and has deacetylase, desuccinylase and long-chain deacylase activities, and plays a key role in various cellular activities (Chen et al., 2016; Li et al., 2016; Tong et al., 2017). Studies have shown that compared with healthy control groups, the expression levels of all Sirtuins in fibroblasts from patients with pulmonary fibrosis tend to decrease, with the most significant decrease in SIRT7 expression. SIRT7 overexpression can decrease TGF-β-induced fibroblast production of collagen and α-smooth muscle actin (α-SMA) (Wyman et al., 2017). SIRT3 is a major deacetylase and plays a crucial role in controlling mitochondrial activity. As a key regulator of mitochondrial activity, SIRT3 exerts its antioxidant stress and maintains mitochondrial homeostasis by regulating the acetylation levels of proteins such as GSK3β, Smad3, SOD2, and IDH2. Deficiency of SIRT3 may lead to alveolar epithelial cell damage, mitochondrial DNA damage, cell senescence, EMT and FMT. SIRT6 is located in the nucleus and has deacetylase, ADP-ribosyltransferase and long-chain dehydrogenase activities (Van Meter et al., 2014; Wang et al., 2016). SIRT6 plays an essential part in DNA damage repair, telomere maintenance, genome stability, inflammatory response and metabolic homeostasis (Kugel and Mostoslavsky, 2014).

**FIGURE 1:**
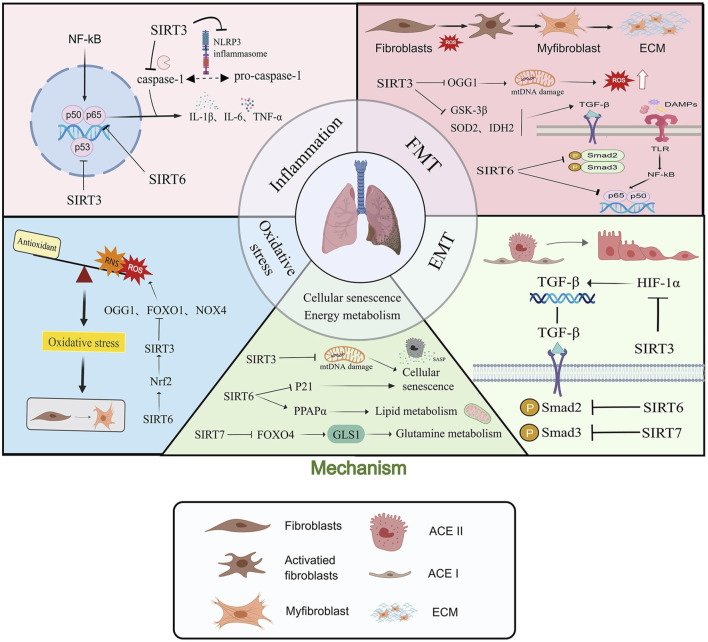
Sirtuins are involved in the development of pulmonary fibrosis by regulating multiple molecular mechanisms such as inflammatory response, oxidative stress, Epithelial-to-mesenchymal transition (EMT), fibroblast-to-myofibroblast transition (FMT), cell senescence and energy metabolism. “→” represents “activation”; “—|” represents “inhibition”.

In summary, In the development of pulmonary fibrosis, sirtuins play a key role by modulating several pathogenic processes, such as inflammatory response, oxidative stress, EMT, FMT, cell death, cell senescence and energy metabolism, and play a vital protective role in the prognosis of fibrosis (Figure 1; Table 1).

**TABLE 1:** List of molecular mechanism of SIRT3/6/7 regulating pulmonary fibrosis.

Phenotype	Molecular	Target	Experimental model	Reference
Inflammation	SIRT3	FOXO1	A549 cells	Zang et al. (2023)
	SIRT6	p65	Primary human keratinocytes	(Kawahara et al., 2009, p. 9)
	SIRT3	NLRP3, caspase-1	Mouse macrophages	Tyagi et al. (2018) and Kurundkar et al. (2019)
Inflammation, ferroptosis, cellular senescence	SIRT3	P53	ATII cells	Li et al. (2023)
FMT	SIRT3	FOXO3a	IMR-90 cells	Rehan et al. (2021)
	SIRT3	GSK3β	Primary fibroblasts	Sundaresan et al. (2015)
	SIRT3	Smad3	Human lung fibroblasts	Sosulski et al. (2017)
	SIRT3	OGG1	IMR-90 cells	Bindu et al. (2017)
	SIRT6	Smad2, P65	Human fetal lung fibroblasts	Zhang et al. (2019b)
EMT	SIRT6	Smad2	BEAS-2B cells	Chen et al. (2019a)
	SIRT6	Smad3	A549 cells	Tian et al. (2017)
	SIRT3	HIF-1α	Mice	Zhang et al. (2019a)
Oxidative stress	SIRT3	SOD2, IDH2	human lung fibroblasts	Sosulski et al. (2017)
	SIRT6	Nrf2	H9c2	Kanwal et al. (2019)
	SIRT3	MnSOD	A549 cells	Guo et al. (2017) and Jablonski et al. (2017)
	SIRT3	PGC1-α	Mouse lung fibroblasts	He et al. (2024a)
	SIRT3	FOXO1	A549 cells	Zang et al. (2023)
	SIRT3	OGG1	Cardiac myocytes	Pillai et al. (2016)
Cellular senescence	SIRT3	PGC1-α	Mouse lung fibroblasts	He et al. (2024a)
	SIRT3	SOD2, OGG1	A549 cells	Zhou et al. (2024)
	SIRT6	P21	HBE cells	
lipid metabolism	SIRT6	PPARα	A549 cells	He et al. (2024b)
Glutamine metabolism	SIRT7	FOXO4	AKR-2B cell	(Choudhury et al., 2020, p. 1)

### 3.1 Inflammation

Inflammation is the body’s immune response to infection or injury and usually plays an important role in maintaining tissue homeostasis in response to harmful stimuli. The inflammatory pathway consists of inducers, sensors, inflammatory mediators, and target tissues (Medzhitov, 2010). Tissue damage and the inflammatory response are key triggers of tissue regeneration and fibrosis (Mack, 2018). Injured tissue not only triggers an inflammatory response, but also regulates the type and direction of inflammation by activating different cell types in the innate and adaptive immune systems (Gao et al., 2019; Mekhlef et al., 2023). A controlled inflammatory response helps prevent infection and further tissue damage, while uncontrolled inflammation maintains autoimmune responses and malignant transformation (Singh et al., 2018).

Inflammatory mediators are chemical substances produced during the inflammatory process. After the initiator triggers the inflammatory response, immune cells expressing sensors such as Toll-like receptors (TLRs) act on target tissues by releasing inflammatory mediators, including vasoactive amines, eicosanoids, pro-inflammatory cytokines, and acute phase proteins. These mediators mediate the inflammatory process by preventing further tissue damage, and ultimately promote tissue regeneration and fibrosis (Abdulkhaleq et al., 2018). Pro-inflammatory cytokines include IL-1, IL-6, IL-8, IL-10, TNF-α, etc. These factors bind to their corresponding receptors, activating intracellular pro-inflammatory signal pathways. These signals further activate TGF-β, STAT3, NF-κB, Hippo pathways, arachidonic acid metabolism and epigenetic pathways, promoting the expression of different proteins. These signals can either eliminate inflammation or, if unchecked, lead to chronic injury, tissue fibrosis and malignant transformation (Chen et al., 2018). Among them, NF-κB is considered to be a core regulatory factor in the inflammatory response, which drives the expression of cytokines, chemokines, inflammatory body components and adhesion molecules. NF-κB is mainly involved in immune responses and inflammatory responses, and can induce the expression of downstream inflammatory cytokines (Koch et al., 2014; Swanson et al., 2019). Studies have shown that SIRT3 is lowly expressed in hyperoxia-induced lung tissue and A549 cells, and that overexpression of SIRT3 can improve the hyperoxia-induced inflammatory response, oxidative stress and apoptosis (Zang et al., 2023). Kawahara et al. found that SIRT6 can deacetylate histone H3 lysine 9 (H3K9) on the promoter of the NF-κB subunit p65 and inhibit the expression of pro-inflammatory cytokines (Kawahara et al., 2009). Furthermore, via blocking the NF-κB signaling pathway, SIRT6 prevents TGF β1-induced differentiation of lung fibroblasts, as demonstrated by other research (Zhang et al., 2017; Tao et al., 2023).

Pulmonary fibrosis is characterized by inflammatory secretion and fibrous hyperplasia, including the overproduction of pro-inflammatory cytokines (such as IL-1β and TNF-α) and transcription factors (such as NF-κB) (Wolters et al., 2014). Long-term overexpression of these inflammatory cytokines can lead to fibroblast proliferation, epithelial cell damage, and excessive deposition of ECM proteins, which in turn damage the alveolar structure (Wilson and Wynn, 2009). Li et al. found that SIRT3 deficiency aggravated the inflammatory response and collagen deposition in lung tissue after PM2.5 treatment, while SIRT3 overexpression reduced iron death, inflammation, and cellular senescence in alveolar epithelial cells by deacetylating P53 (Li et al., 2023). In addition, SIRT3 agonists (such as melatonin) can also alleviate PM2.5-induced aging and ferroptosis in mice, and targeting the USP3-SIRT3-P53 axis may provide new strategies for the treatment of pulmonary fibrosis (Li et al., 2023).

### 3.2 Fibroblast-to-myofibroblast transition

A key step in pulmonary fibrosis is the TGF-β1-mediated FMT. FMT is considered to be the major source of myofibroblast accumulation (Hinz et al., 2012; Tsubouchi et al., 2017). There is mounting evidence that FMT is essential to the beginning and progression of pulmonary fibrosis (Han et al., 2021b; Liu et al., 2021; Wang et al., 2021a). TGF-β1 and other stressors activate lung fibroblasts under pathological situations, raising intracellular ROS levels that promote FMT and eventually result in IPF (El Agha et al., 2017). Consequently, FMT is an essential target for IPF treatment.

Current research shows that members of the Sirtuins family can block the FMT process, thereby reducing lung fibrosis. Rehan et al. found that in a mouse model of bleomycin-induced lung injury, restoring SIRT3 expression with a cDNA overexpression plasmid can significantly reduce established lung fibrosis by attenuating FMT (Rehan et al., 2021). In addition, SIRT3 also regulates the transformation of fibroblasts into myofibroblasts by deacetylating glycogen synthase kinase 3β (GSK3β) at K15 and inhibiting the TGF-β1 signaling pathway, which promotes fibrosis (Sundaresan et al., 2015). Sosulski et al. found in in vivo experiments that SIRT3-deficient mice are more likely to develop lung fibrosis, while *in vitro* experiments showed that reduced expression of SIRT3 in lung fibroblasts promoted FMT. This may be due to the fact that the reduction of SIRT3 leads to the blockage of the deacetylation and activation of SOD2 (K68) and IDH2 (K143), thereby reducing the efficiency of the oxidative stress response and promoting the occurrence of FMT. In contrast, overexpression of SIRT3 can inhibit TGF-β1-mediated FMT and significantly reduce the level of SMAD3 (Sosulski et al., 2017). In addition, it has been found that SIRT3 inhibits FMT by regulating the acetylation level of OGG1 to reduce mitochondrial DNA damage in fibroblasts (Bindu et al., 2017; Mora et al., 2017). In addition, SIRT6 has been shown to prevent lung fibroblasts from differentiating into myofibroblasts by blocking the NF-κB signaling pathway which is triggered by TGF-β1 (Zhang et al., 2017; Tao et al., 2023). Upon TGF-β1 stimulation, SIRT6 inhibited NF-κB-dependent transcriptional activity by decreasing the phosphorylation level of Smad2 and its nuclear translocation. In addition, SIRT6 interacted with the NF-κB subunit p65, further inhibiting the TGF-β1-induced NF-κB pathway (Zhang et al., 2019b).

In general, SIRT3 inhibits the occurrence of FMT by regulating the acetylation levels of proteins such as GSK3β, Smad3, SOD2, and IDH2, while SIRT6 inhibits TGF-β1-induced related signal pathways by reducing the phosphorylation and nuclear translocation of Smad2.

### 3.3 Epithelial-to-mesenchymal transition

EMT is a key cellular process in the process of tissue fibrosis (Lamouille et al., 2014). TGF-β1 is a major inducer of ECM production, and its activation accelerates the process of EMT in epithelial cells, promoting the generation of fibroblasts and the accumulation of ECM proteins in tissues (Lamouille et al., 2014). When pulmonary fibrosis develops, EMT is thought to be a reversible process in which epithelial cells eventually lose the epithelial cell adhesion protein E-cadherin (E-cad) and gain mesenchymal traits including the mesenchymal marker α-smooth muscle actin (α-SMA) (Bartis et al., 2014; Qu et al., 2019). Excessive proliferation of cells that produce extracellular matrix results from an imbalance between the alveolar epithelium and the mesenchymal cells that line it (Barkauskas and Noble, 2014, p. 7).

EMT is generally considered to be another major source of myofibroblasts (Lamouille et al., 2014). Chen et al. showed that SIRT6 can inhibit EMT in human bronchial epithelial cells (BEAS-2B) by participating in the TGF-β1 signaling pathway and inhibiting lung fibrosis. Overexpression of SIRT6 can inactivate the TGF-β1/Smad2 signaling pathway (Chen et al., 2019a). In addition, SIRT6 can further inhibit EMT by inhibiting the TGF-β1/Smad3 signaling pathway and the Smad3-Snail1 complex and EMT-related transcription factors. Adeno-associated virus delivery of SIRT6 can significantly reduce bleomycin-induced alveolar epithelial cell damage and lung fibrosis Zhang et al. found that restoring SIRT3 expression enhances its activation of HIF-1α, which in turn suppresses TGF-β1 levels, thereby improving EMT and pulmonary fibrosis (Zhang et al., 2019a).

### 3.4 Oxidative stress

Oxidative stress is a significant factor in the onset and pathophysiology of IPF and is closely related to the progression of IPF. An imbalance between ROS, reactive nitrogen species (RNS) and antioxidant defenses can lead to oxidative stress, which in turn can cause cell dysfunction and tissue damage (Reddy et al., 2009). Increased oxidative stress appears to induce premature cellular senescence, which prompts fibroblasts to acquire resistance to apoptosis and maintain metabolic activity, ultimately producing high levels of reactive oxygen species (ROS), which contributes to the development of FMT (Toussaint et al., 2000; Dasari et al., 2006; Campisi and d’Adda di Fagagna, 2007). Studies have shown that oxidative stress promotes the transformation and proliferation of lung fibroblasts by releasing a variety of cytokines (Han et al., 2014). Clinical studies have found that compared with the control group, the levels of lipid peroxides and apoptosis of lung epithelial cells in patients with pulmonary fibrosis are significantly higher, and the level of oxidative stress is positively correlated with the degree of fibrosis (Molyneaux and Maher, 2013). Matsuzawa et al. found that there is a general oxidative/antioxidative imbalance and increased oxidative stress in patients with IPF by measuring oxidative stress markers in serum (Huang et al., 2017).

During the process of pulmonary fibrosis, members of the Sirtuins family are involved in regulating oxidative stress. Studies have found that SIRT6 and SIRT3 have a synergistic effect, with the two regulating each other: SIRT3 suppresses oxidative stress, while SIRT6 further activates the expression of SIRT3 through increasing nuclear factor erythroid 2-related factor 2 (Nrf2)-dependent transcription (Kanwal et al., 2019). NOX4-dependent hydrogen peroxide (H2O2) production plays a key role in TGF- β1-induced myofibroblast differentiation, ECM production, and contraction. NOX4 promotes the migration and differentiation of fibroblasts to myofibroblasts through the ALK5/Smad3 upstream signaling pathway by increasing mitochondrial ROS production, promoting alveolar epithelial cell (AEC2) death and impairing mitochondrial function (Hecker et al., 2009). Studies have shown that SIRT3 deficiency is more common in IPF patients, and SIRT3 knockout (SIRT3−/−) mice exhibit significantly exacerbated pulmonary fibrosis. *In vitro* experiments have shown that SIRT3 detoxifies mitochondrial reactive oxygen species by deacetylating manganese superoxide dismutase (MnSOD) at the K68 site and mitochondrial 8-oxoguanine DNA glycosylase (OGG1) at the K338/341 site, while decreased SIRT3 expression may lead to elevated mitochondrial ROS levels and mitochondrial DNA (mtDNA) damage through the OGG1-MnSOD-SIRT3 axis, thereby exacerbating apoptosis of alveolar epithelial cells (Guo et al., 2017; Jablonski et al., 2017). In contrast, overexpression of SIRT3 can ameliorate oxidant-induced AEC mtDNA damage (Cheresh et al., 2021). In addition, He et al. found that activating the PGC-1α/SIRT3 pathway can exert an anti-oxidative stress effect, thereby inhibiting fibroblast senescence and slowing the progression of pulmonary fibrosis (He et al., 2024a). Zang et al. found that SIRT3 overexpression improves hyperoxia-induced inflammation, oxidative stress and apoptosis by regulating the acetylation level of FOXO1 (Zang et al., 2023).

OGG1 (oxygen-base guanine glycosylase) is involved in the hydrolysis of 8-oxo-dG in DNA. 8-oxo-dG is an oxidized guanine residue in DNA. Defects in OGG1 can lead to mitochondrial DNA damage (de Souza-Pinto et al., 2001). Pillai VB et al. found that SIRT3 as an endogenous negative regulator of pulmonary fibrosis, its overexpression can attenuate TGF-β1-induced mtDNA damage in pulmonary fibroblasts. SIRT3 prevents the degradation of OGG1 by binding to it and deacetylating it, thereby preventing the accumulation of 8-oxo-dG adducts. This mechanism was verified in in vivo experiments. Studies have shown that SIRT3 deficiency exacerbates bleomycin-induced fibrosis in mice, while SIRT3 transgenic mice exhibit significantly reduced fibrosis (Pillai et al., 2016). In addition, According to earlier research, SIRT3-deficient mice are more vulnerable than wild-type mice to asbestos-induced lung fibrosis and mtDNA damage (Cheresh et al., 2021). These results suggest that SIRT3 regulates mtDNA repair, which may be an important mechanism involved in the development of lung fibrosis.

Current research suggests that sirtuins play a key role in oxidative stress, mainly related to the following proteins or genes: NF-κB, Nrf2, the FOXO family, PGC-1α, p53 and AMPK (Wu et al., 2022).

### 3.5 Cellular senescence and energy metabolism

Cellular senescence contributes to the development of IPF through multiple mechanisms, including the senescence-associated secretory phenotype (SASP) (Blokland et al., 2020), abnormal telomere function (Lee et al., 2021), mitochondrial dysfunction (Sullivan et al., 2021), DNA damage (Schuliga et al., 2020), epigenetic changes (Wang et al., 2021b), inflammatory responses (Tao et al., 2021) and protein homeostasis imbalance (Sellares et al., 2019). Studies have found that the alveolar epithelial cells of IPF patients show significant signs of aging, such as mitochondrial abnormalities, dysfunction, and shortened telomeres (Minagawa et al., 2011; Bueno et al., 2015). The pro-fibrotic mediators secreted by senescent epithelial cells, such as IL-1β, IL-6, and IL-8, encourage fibroblasts to differentiate into myofibroblasts and increase their resistance to apoptosis, which in turn leads to the accumulation of myofibroblasts and extracellular matrix, ultimately forming fibrotic lumps (Minagawa et al., 2011; Chen et al., 2019b; Tian et al., 2019). Among these processes, impaired autophagy, mitochondrial dysfunction and mitochondrial DNA damage are regulated by the Sirtuin family. By regulating these processes, Sirtuins help slow down cellular aging and thus reduce the occurrence of fibrosis.

SIRT3, a mitochondrial deacetylase, can regulate mitochondrial DNA damage in alveolar epithelial cells and lung diseases (Kim et al., 2015; Bindu et al., 2017; Cao et al., 2021). Studies have shown that specific silencing of the Sirt3 gene can inhibit the aging of fibroblasts, and this inhibitory effect is mediated through the PGC1-alpha/Sirt3 pathway (He et al., 2024a). In contrast, activation of Sirt3 can effectively reduce alveolar epithelial cell aging and alleviate mtDNA damage, possibly by activating the antioxidant capacity of SOD2 and the DNA repair function of OGG1 (Zhou et al., 2024). These results suggest that targeting Sirt3, the PGC1-alpha/Sirt3 pathway, SOD2 and OGG1 may be potential strategies for the treatment of pulmonary fibrosis. In addition, it has been found that the expression of senescence markers is increased in IPF lung samples, and that overexpression of SIRT6 can inhibit TGF-β-induced HBE cell senescence by promoting the degradation of the p21 protein (Minagawa et al., 2011). In IPF lungs and the lungs of aged mice, downregulation of zinc transporter ZIP8/SLC39A8 is associated with alveolar epithelial cell senescence and exacerbation of pulmonary fibrosis. Exogenous zinc supplementation and activation of SIRT1 promote the self-renewal and differentiation of AT2 cells in the lungs of IPF patients and aged mice, thereby reducing pulmonary fibrosis (Liang et al., 2022).

In terms of energy metabolism, He et al. found that SIRT6 alleviates bleomycin-induced lipotoxicity by encouraging the breakdown of lipids, which raises the energy supply and decreases lipid peroxide levels, thereby slowing the development of pulmonary fibrosis (He et al., 2024b). Choudhury found that SIRT7 regulates glutamine metabolism through the TGF-β/SIRT7/FOXO4 axis, promotes the deacetylation of FOXO4, thereby inhibiting the expression of GLS1, and thus controls the progression of pulmonary fibrosis (Choudhury et al., 2020, p. 1). In summary, targeting the SIRT6-PPARα-mediated lipolysis and the TGF-β/SIRT7/FOXO4-regulated glutamine metabolic pathway may be a potential strategy for the treatment of pulmonary fibrosis and related diseases.

### 3.6 Cell death

Programmed cell death plays a critical role in maintaining homeostasis and responding to environmental stimuli. When cells are stimulated by external stimuli or stressed by internal factors, they can initiate a protective suicide process, which is regulated by a variety of cell signaling molecules. A variety of programmed cell death modes have been identified, including apoptosis, pyroptosis, necroptosis, autophagy, ferroptosis, cuproptosis, and PANapoptosis (Lockshin, 2016, p. 50). Among them, pyroptosis is a form of programmed cell death driven by inflammasomes, which is characterized by the release of proinflammatory factors along with cell lysis, thereby triggering a strong inflammatory response (He et al., 2015). There is increasing evidence that pyroptosis plays a critical role in the development and progression of PF (Liang et al., 2020; Wei et al., 2023). In a mouse model of PF, the levels of caspase-1 and IL-1β in bronchoalveolar lavage fluid (BALF) were significantly elevated (Li et al., 2018). Studies of a bleomycin-induced PF model showed that mice lacking NLRP3, ASC, or caspase-1 had reduced IL-1β secretion from the lungs, accompanied by a decrease in the degree of inflammation and fibrosis (Stout-Delgado et al., 2016; Wei et al., 2023). Therefore, inhibiting inflammasome activation may be a potential therapeutic strategy for PF. Studies have shown that upregulation of SIRT3 can inhibit pyroptosis by inhibiting the activation of NLRP3 and caspase-1 (Chen et al., 2013; Tyagi et al., 2018; Kurundkar et al., 2019). In addition, vitamin D3 (VD3) may alleviate PF through SIRT3-mediated pyroptosis inhibition (Tang et al., 2023). It is worth noting that the STING signaling pathway can promote the release of IL-1β and IL-18 by inducing the classical activation mode of NLRP3 inflammasomes through K^+^ efflux and membrane perturbation, thereby driving aseptic inflammation and pyroptosis (Oduro et al., 2022). Zhou et al. further revealed that SIRT3 enhances the activity of superoxide dismutase 2 (SOD2) by deacetylation, thereby regulating the cGAS/STING and NF-κB signaling pathways, maintaining mitochondrial DNA integrity, and thereby inhibiting the progression of pulmonary fibrosis and mitochondrial damage (Zhou et al., 2024). These studies suggest that SIRT3 may play a role in pyroptosis regulation by targeting the NLRP3, caspase-1, cGAS/STING, and NF-κB signaling pathways, providing new potential intervention targets for the treatment of PF.

Apoptosis, a form of irreversible cell death, was proposed by Kerr and colleagues in 1972 and is characterized by a cascade of events triggered by the activation of cysteine aspartate-specific proteases (caspases) (Kerr et al., 1972). Morphologically, apoptotic cells show cell shrinkage, volume reduction, nuclear condensation and nuclear fragmentation, but the cell membrane remains intact (Medina et al., 2020). Studies have shown that oxidative stress-induced mitochondrial DNA (mtDNA) damage promotes apoptosis of AEC cells and lung fibrosis (Kim et al., 2015). Renea P Jablonski et al. found that SIRT3 deficiency can exacerbate mtDNA damage and apoptosis of alveolar epithelial cells, thereby promoting the progression of lung fibrosis (Jablonski et al., 2017). In addition, airway delivery of SIRT3 cDNA significantly improved pulmonary fibrosis in aged mice. This effect was associated with activation of the forkhead transcription factor FoxO3a in fibroblasts, which in turn regulated the expression of Bcl-2 family pro-apoptotic proteins and enhanced the sensitivity of fibroblasts to apoptosis (Rehan et al., 2021). These studies suggest that SIRT3 may play an important regulatory role in the process of apoptosis by reducing oxidative stress levels and protecting mtDNA integrity. Necroptosis is a novel type of programmed cell death that was first reported by Vercammen and colleagues in 1998. It has a typical necrotic morphology, but its upstream molecular regulatory mechanism is similar to that of apoptosis (Vercammen et al., 1998). Among them, receptor-interacting protein kinase 1 (RIPK1), RIPK3 and mixed lineage kinase domain-like protein (MLKL) are key regulatory factors (Samson et al., 2020). In various disease models, SIRT3 deficiency is closely related to the activation of necroptosis. Studies have shown that SIRT3 deficiency can significantly upregulate necroptosis-related proteins, including RIPK1, RIPK3, and caspase-3, and increase the expression of the inflammation-related proteins NLRP3, caspase-1, and IL-1β, thereby exacerbating mitochondrial damage and oxidative stress, and ultimately promoting necroptosis (Yang et al., 2020; Song et al., 2021). These findings suggest that SIRT3 may be involved in the regulation of necroptosis by regulating the expression of RIPK1, RIPK3 and caspase-3, providing a new potential target for intervention in related diseases.

**FIGURE 2:**
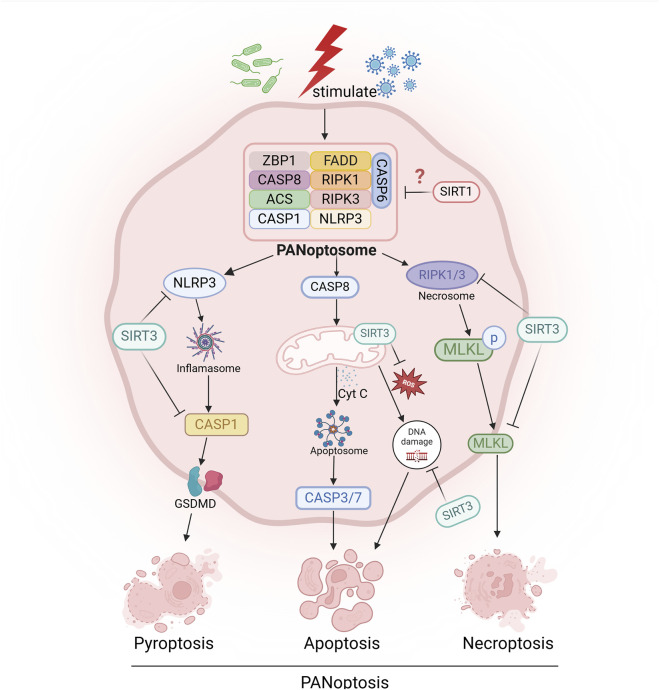
Sirtuins are involved in the regulation of programmed cell death, such as pyroptosis, apoptosis, necroptosis, and PANoptosis. “→” represents “activation”; “—|” represents “inhibition”.

PANoptosis is a novel form of inflammatory cell death regulated by the PANoptosome complex, which combines key features of pyroptosis, apoptosis and necroptosis (Samir et al., 2020). This mode of cell death is typically associated with the assembly of the PANoptosome, a multiprotein complex that is essential for the induction of pathogen-associated molecular patterns (PAMPs), damage-associated molecular patterns (DAMPs) and other risk factors. The components of the PANoptosome vary depending on the stimulus, but typically include Z-DNA binding protein 1 (ZBP1), receptor-interacting protein kinase 3 (RIPK3), receptor-interacting protein kinase 1 (RIPK1), apoptosis-related speck-like protein containing a caspase recruitment domain (ASC), Fas-associated protein with death domain (FADD), and caspase-8 (CASP8) (Zhu et al., 2023) In addition, the PANoptosome also contains key molecules that mediate pyroptosis, apoptosis and necroptosis, and is a core entry point for studying the mechanism and regulatory strategies of PANoptosis (Zhu et al., 2023). Recent studies have shown that SIRT1 may play a protective role in the regulation of PANoptosis. For example, lycopene can inhibit monensin B1-induced mitochondrial damage and PANoptosis through a SIRT1-dependent mechanism, suggesting a key regulatory role of SIRT1 in cell survival and energy metabolism (Wang et al., 2025). Similarly, it was found that vagal nerve stimulation (VNS) can inhibit chemotherapy-induced PANoptosis and promote neurological recovery through a SIRT1-dependent pathway (Tang et al., 2025). These studies further suggest that SIRT1 may be a potential target for intervention in diseases related to PANoptosis, providing new ideas for the development of subsequent treatment strategies (Figure 2).

## 4 Targeting Sirt3/6/7 in therapeutic strategies for pulmonary fibrosis

Since the Sirtuins family was identified as a potential drug target, many pharmacological and pharmaceutical studies have focused on the development of natural and synthetic Sirtuin regulators. Among these studies, honokiol (HNK), a novel lignin active ingredient, has been shown to be a natural inducer of SIRT3. Zhou et al. showed that HNK significantly alleviates the aging of alveolar type II epithelial cells (ATII cells) by activating SIRT3, which in turn enhances the activity of SOD2 activity, regulated the cGAS/STING and NF-κB signaling pathways, significantly alleviated the aging of ATII cells, protected mitochondrial DNA integrity, and thus effectively prevented pulmonary fibrosis and mitochondrial damage (Zhou et al., 2024). In addition, resveratrol, a polyphenolic compound, has been found to inhibit the differentiation of myofibroblasts and promote the transcriptional expression of SIRT3 (Olson et al., 2005; Zhou et al., 2014; Chen et al., 2015b). Sosulski et al. showed that resveratrol improved the acetylation changes of SOD2 and IDH2 induced by TGF-β1 by promoting the expression of SIRT3 (Sosulski et al., 2017). Similarly, cryptotanshinone is also considered an effective drug for the treatment of pulmonary fibrosis because it can regulate the TGF-β1/Smad3, STAT3 and SIRT3 signaling pathways (Wang et al., 2022).

Research on other natural compounds has also provided strong evidence for the treatment of IPF. For example, Ji-Hong et al. found that baicalein can inhibit the downregulation of Sirt3 expression induced by bleomycin in the lung, thereby inhibiting the TGF-β1/Smad signaling pathway and lung fibrosis, providing a novel experimental basis for the treatment of IPF (Ji-Hong et al., 2023). Similarly, Fu et al. found that mangiferin effectively alleviates diabetes-induced pulmonary fibrosis by enhancing AMPK activity, promoting the phosphorylation of FoxO3 and the expression of SIRT3(Fu et al., 2024). Tang et al. showed that vitamin D3 (VD3) intervention may inhibit the activation of the caspase-1/GSDMD-N and caspase-3/GSDME-N pathways by increasing SIRT3 expression, thereby inhibiting pyroptosis and alleviating pulmonary fibrosis (Tang et al., 2023). In addition, some scholars have found that Hirudin can inhibit fibroblast senescence by promoting the expression of PGC1-α and SIRT3 in fibroblasts, activate the PGC1-α/SIRT3 pathway, and effectively reduce the levels of ROS and oxidative stress markers, thereby exerting an anti-pulmonary fibrosis effect (He et al., 2024a). Zhang et al. found that Probusco can restore the expression level of SIRT3, reduce the production of TGF-β1 and EMT induced by bleomycin, and at the same time inhibit oxidative stress, thereby alleviating pulmonary fibrosis (Zhang et al., 2019a).

In addition, natural products such as fisetin, quercetin, kaempferol, punicalagin, taxifolin and caffeic acid may also act as Sirtuins activators. These compounds may be involved in physiological processes such as mitochondrial energy metabolism, oxidative stress regulation and anti-aging by activating the expression of the SIRT3/6 gene (Grozinger et al., 2001; Houtkooper et al., 2010; Hubbard and Sinclair, 2014) (Table 2).

**TABLE 2:** List of SIRT3/6/7 regulators in pulmonary fibrosis.

Compound	Target	Action Path	Reference
Cryptotanshinone	SIRT3	Regulation of TGF-β1/Smad3, STAT3 and SIRT3 pathways	Wang et al. (2022)
Resveratrol	SIRT3	Promoting SIRT3 expression and ameliorating TGF-β1-induced changes in SOD2 and IDH2 acetylation	Sosulski et al. (2017)
Scutellarin	SIRT3	Restoration of SIRT3 expression, thereby inhibiting the TGF-β1/Smad pathway	Ji-Hong et al. (2023)
Mangiferin	SIRT3, AMPK, FOXO3	Directly enhanced AMPK activity and upregulated FoxO3 phosphorylation and SIRT3 expression level	Fu et al. (2024)
Probucol	SIRT3	Restoration of SIRT3 expression, reduction of its HIF-1α activation and TGF-β1 release to improve EMT	Zhang et al. (2019a)
VD3	SIRT3	Increased SIRT3 expression to inhibit cellular pyroptosis	(Tang et al., 2023, p. 2)
Hirudin	PGC1-α, SIRT3	Activation of the PGC1-alpha/Sirt3 pathway inhibits fibroblast senescence	He et al. (2024a)
Honokiol	SIRT3	Regulates cGAS/STING, NF-κB signaling pathways and effectively attenuates senescence and mtDNA damage in ATII cells	Zhou et al. (2024)

Understanding the upstream regulatory mechanisms of sirtuin expression and activity is crucial for revealing their role in physiological and pathological processes and developing therapeutic strategies targeting SIRT3/6/7. The expression and activity of sirtuins are regulated at multiple levels, among which microRNAs (miRNAs) act as key post-transcriptional regulatory factors that affect the mRNA stability and protein expression levels of sirtuins. For example, miR-23b-3p, miR-494-3p, miR-224 and miR-421 reduce SIRT3 expression by inhibiting mRNA translation or promoting mRNA degradation (Kadota et al., 2020; Chen and Dai, 2023; Chen et al., 2024a); transcription factors SNAI1 and ZEB1 inhibit SIRT3 promoter activity (Zhang et al., 2020); and ZMAT1 promotes transcription by binding to the SIRT3 promoter (Ma et al., 2022). Similarly, miR-34a-5p, miR-486-3p, miR-122 and miR-26a target SIRT6 and inhibit its expression (Kaitsuka et al., 2021; Chen et al., 2024b; Li et al., 2024b; Mal et al., 2024); the transcription factor FOXO3a binds to the SIRT6 promoter and enhances its expression (Dong et al., 2020). In addition, studies have shown that miR-21-5p promotes tumor progression by regulating the ubiquitination of SIRT7 (Hu et al., 2023); while miR-148b-3p, miR-770 and PYCR1 protein target SIRT7 to inhibit its expression or transcription (Sun et al., 2018; Zheng et al., 2023; Li et al., 2024a). These regulatory factors not only affect the transcription and translation of Sirtuin, but also may regulate its function in metabolism, inflammatory response and fibrotic diseases. Further research into these mechanisms will provide a theoretical basis for the development of Sirtuin-related therapeutic strategies and enhance its application potential in disease intervention.

## 5 Clinical evidence and challenges

We found 36 clinical trials on SIRT3, SIRT6 and SIRT7 in databases such as PubMed, ClinicalTrials.gov, the WHO International Clinical Trials Registry Platform (ICTRP), the Chinese Clinical Trial Registry (ChiCTR) and the ISRCTN Registry. Among these, 31 studies focused on the effects of different interventions on the expression of SIRT3, SIRT6 and SIRT7 proteins in human samples, while five studies evaluated the effects of SIRT activators (such as resveratrol, curcumin, SP-624 and IMU-856) and SIRT inhibitors (nicotinamide) on the physiological functions of subjects (Table 3). In a retrospective randomized controlled clinical trial, the researchers analyzed blood samples from 30 patients with acute coronary syndrome (ACS) to detect the levels of the inflammatory factors IL-1β, IL-18, TnI, and CK-MB and the expression of SIRT6. The results showed that Sirt6 can prevent or inhibit the development of ACS (Tian and Zhang, 2024). In addition, IMU-856, a small molecule SIRT6 regulator, was shown to be safe and well tolerated in a clinical trial in celiac disease patients, supporting once-daily dosing, and to improve relevant pharmacodynamic parameters during gluten exposure, suggesting that SIRT6 may be a potential therapeutic target (Daveson et al., 2025). Similarly, two phase I studies evaluated the pharmacokinetics and safety of SIRT6 activator SP-624 in healthy adults. The results showed that single and multiple doses of SP-624 were safe and well tolerated. A daily dose of 20 mg is currently being used in a phase II clinical trial to treat major depressive disorder (MDD) (Rigdon et al., 2025). In addition, a study by Javier Traba et al. found that 24-h fasting activates SIRT3 and may improve the body’s tolerance to NLRP3 inflammasome activation by reducing mitochondrial ROS levels (Traba et al., 2015). A retrospective study of 127 Chinese patients with colon cancer found that high expression of SIRT3 in the cytoplasm was significantly associated with high tumor grade, positive lymph node status, and poor prognosis, suggesting that SIRT3 may support tumor cell survival by promoting cell proliferation, migration, and invasion. Therefore, protein therapy targeting SIRT3 may be used as a complementary or alternative strategy to existing colon cancer chemotherapy (Liu et al., 2014). In summary, existing studies support the feasibility of clinical trials and treatment strategies targeting SIRT3 and SIRT6, providing a theoretical basis for the further development of Sirtuin-targeted therapies for pulmonary fibrosis.

**TABLE 3:** List of clinical trials on SIRT3/6/7.

ID	Molecular	Year
NCT06617988	SIRT3	2024
ChiCTR2300074357	SIRT3	2024
NCT05249309	SIRT3	2021
CTRI/2021/05/033,485	SIRT3	2021
ChiCTR2000033555	SIRT3	2020
ITMCTR1900002787	SIRT3	2019
ChiCTR1800016122	SIRT3	2018
ChiCTR-COC-15007025	SIRT3	2015
NCT05808387	SIRT3	2023
NCT02812238	SIRT3	2019
ChiCTR1900027798	SIRT3	2019
NCT06337227	SIRT6	2024
NCT05417061	SIRT6	2023
NCT01508091	SIRT6	2012
NCT02011906	SIRT3, 6	2013
JPRN-jRCT1012240016	SIRT6	2024
JPRN-UMIN000049014	SIRT6	2022
ChiCTR2000039808	SIRT6	2020
IRCT20180912041018N2	SIRT6	2019
NCT01567176	SIRT6	2012
NCT04303208	SIRT7, 3	2022
ChiCTR1800017278	SIRT7	2018
ChiCTR1800017244	SIRT7	2018
Wasserfurth et al. (2021)	SIRT3	2021
Oh et al. (2024)	Sirtuins	2024
Cho et al. (2022)	SIRT3	2022
Bańkowski et al. (2022)	SIRT3	2022
Traba et al. (2015)	SIRT3	2015
Lilja et al. (2021)	SIRT3	2021
Liu et al. (2014)	SIRT3	2014
Hooshmand-Moghadam et al. (2020)	SIRT3, 6	2020
Nikooyeh et al. (2021)	SIRT6	2021
Daveson et al. (2025)	SIRT6	2025
Tian and Zhang (2024)	SIRT6	2024
González-Fernández et al. (2019)	SIRT3, 6, 7	2019
Rigdon et al. (2025)	SIRT6	2025

As research on SIRT regulators intensifies, more and more of these drugs are entering the clinical trial stage, but at the same time, they face many challenges. These challenges mainly involve target specificity, drug delivery, pharmacokinetic properties, the complexity of biological functions, and the limitations of preclinical research. The Sirtuins family has a total of seven members (SIRT1-7), and their highly similar structures make it extremely challenging to develop drugs that specifically target SIRT3, SIRT6, or SIRT7. Non-specific effects may affect efficacy and increase side effects (Han et al., 2021a; Zhang et al., 2022). In addition, since these proteins are mainly located in the nucleus or mitochondria, how to accurately deliver drugs to the corresponding subcellular structures while optimizing pharmacokinetic properties to improve efficacy and reduce side effects remains a key issue to be solved (Han et al., 2021a). SIRT3, SIRT6 and SIRT7 have diverse biological functions in different tissues and cell types. Therefore, a deeper understanding of their mechanisms of action in different physiological and pathological states is critical to ensure the safety and efficacy of targeted therapies. However, current research is mostly at an early stage, and there is a lack of large-scale preclinical and clinical data, which limits the comprehensive evaluation and clinical translation of SIRT proteins as therapeutic targets. There are still many limitations, such as the differences between animal models and human diseases, the differences in the functions of SIRT proteins in different species, individual differences, the complex pathophysiological mechanisms of PF diseases, and long-term effects. For example, commonly used animal models (such as the bleomycin-induced pulmonary fibrosis model) cannot completely reproduce the complex pathological characteristics of human pulmonary fibrosis, which may lead to poor efficacy of therapeutic strategies that perform well in animal models in human trials (Song et al., 2022). In addition, the differences in the functions of SIRT proteins in different species may affect the feasibility of their clinical application.

Currently, specific agonists or inhibitors targeting SIRT3, SIRT6 and SIRT7 are still limited, and most studies are short-term observations. There is a lack of systematic evaluation of the long-term effects and potential side effects of their use. Their safety and efficacy have not been fully verified in humans, which limits the advancement from preclinical studies to clinical trials. In the future, long-term clinical trials will need to be conducted to systematically evaluate the long-term safety and efficacy of Sirtuin modulators. In addition, the occurrence and development of pulmonary fibrosis are affected by multiple factors such as genetic background, environmental exposure and coexisting diseases (King et al., 2011), and current preclinical studies have difficulty comprehensively simulating these complex factors, which may lead to results in human trials that do not match expectations. Therefore, in future research, animal models need to be optimized to improve the specificity and delivery efficiency of Sirtuin-targeted drugs, while combining genetics, omics analysis and precision medicine strategies to promote the clinical application of SIRT3, SIRT6 and SIRT7 targeted therapies.

Although clinical therapies targeting SIRT3, SIRT6 and SIRT7 still face challenges in terms of drug specificity, pharmacokinetics and the complexity of biological functions, the development of new compounds and in-depth research on their mechanisms give them good prospects for future application. However, in the process of translation into human clinical trials, limitations such as safety and toxicity assessment, differences between animal models and human diseases, individual variation and long-term efficacy still need to be overcome. Therefore, clinical trial protocols should be designed carefully, taking these potential factors into full consideration, and systematic safety and toxicity studies should be carried out to ensure that the therapy will not adversely affect healthy tissues.

## 6 Conclusion and outlook

The sirtuin family is an important class of deacetylases that are widely involved in the regulation of cellular metabolism, stress response, aging, and various diseases. The sirtuin family, especially the subtypes SIRT1, SIRT3, SIRT6, and SIRT7, plays a key role in the study of IPF. These sirtuin subtypes exhibit significant antifibrotic effects by regulating oxidative stress, inflammatory response, apoptosis, EMT, and fibrosis-related signaling pathways. Although a large number of studies have revealed the potential protective effects of Sirtuins in pulmonary fibrosis, the precise regulation of Sirtuins activity to achieve the desired therapeutic effect remains an important challenge.

Natural compounds, synthetic small molecules, and gene editing strategies provide potential therapeutic approaches for the treatment of pulmonary fibrosis. In particular, the specific activation or inhibition of SIRT3, SIRT6, and SIRT7 may open up new directions for the treatment of pulmonary fibrosis. This article reviews the role of SIRT3, SIRT6 and SIRT7 in lung fibrosis, which are relatively understudied in the Sirtuins family. It analyzes their mechanisms of action in detail and discusses therapeutic strategies targeting SIRT3, SIRT6 and SIRT7 and their related molecular pathways, providing useful clues for the future search for new IPF treatment targets and the development of new therapies. Future treatment strategies may no longer rely on the regulation of a single target, but rather through the combined intervention of multiple pathways to regulate multiple key mechanisms of lung fibrosis. In addition, further optimizing the safety, efficacy and precision of Sirtuins targeted therapies and exploring the specific mechanisms of action of different subtypes will provide more options and strategies for clinical treatment.

In short, the Sirtuins family has shown great potential for the treatment of pulmonary fibrosis. As research progresses, our understanding of its mechanism will be further deepened in the future, which will promote Sirtuins as a new and effective treatment. Although there are still some challenges, by overcoming the limitations of existing research and promoting the clinical application of Sirtuins targeted therapy, it will surely bring new treatment hope to IPF patients. Combined with multi-target therapy and early diagnosis, Sirtuins targeted therapy is expected to become an effective weapon against pulmonary fibrosis.
